# The role of rhIGF-1/BP3 in the prevention of pulmonary hypertension in bronchopulmonary dysplasia and its underlying mechanism

**DOI:** 10.1186/s12890-023-02498-1

**Published:** 2023-06-15

**Authors:** Sehua Qu, Lianqiang Shan, Xin Chen, Zhen Zhang, Yumeng Wu, Yun Chen, Feixiang Zhuo, Yitong Wang, Huaifu Dong

**Affiliations:** 1 Department of Neonatology, The First Affiliated Hospital of Bengbu Medical College, Bengbu, 233000 China; 2 Department of Radiology, The First Affiliated Hospital of Bengbu Medical College, Bengbu, 233000 China; 3 Department of Pediatrics, The First Affiliated Hospital of Bengbu Medical College, Bengbu, 233000 China; 4 Bengbu Medical College, Bengbu, 233000 China

**Keywords:** rhIGF-1/BP3, Angiogenesis, Bronchopulmonary dysplasia, Pulmonary hypertension

## Abstract

**Background:**

This study aimed to determine whether postnatal treatment with recombinant human IGF-1 (rhIGF-1)/binding peptide 3 (BP3) ameliorates lung injury and prevents pulmonary hypertension (PH) in bronchopulmonary dysplasia (BPD) models.

**Methods:**

We used two models of BPD in this study: one model that was associated with chorioamnionitis (CA), stimulated by intra-amniotic fluid and exposure to lipopolysaccharide (LPS), whereas the other was exposed to postnatal hyperoxia. Newborn rats were treated with rhIGF-1/BP3 (0.2 mg/Kg/d) or saline via intraperitoneal injection. The study endpoints included the wet/dry weight (W/D) ratio of lung tissues, radial alveolar counts (RACs), vessel density, right ventricular hypertrophy (RVH), lung resistance, and lung compliance. Hematoxylin and eosin (H&E) and Masson staining were used to evaluate the degree of lung injury and pulmonary fibrosis. IGF-1 and eNOS expression were detected using western blotting or quantitative reverse transcriptase polymerase chain reaction (qRT-PCR). The levels of SP-C, E-cadherin, N-cadherin, FSP1, and Vimentin in the lung tissues were detected by immunofluorescence.

**Results:**

LPS and hyperoxia treatment increased lung injury and pulmonary fibrosis, enhanced RVH and total respiratory resistance, and decreased RAC, pulmonary vascular density and pulmonary compliance in young mice (all p < 0.01). Simultaneously, LPS and hyperoxia induced an increase in epithelial-mesenchymal transition (EMT) in airway epithelial cells. However, rhIGF-1/BP3 treatment reduced lung injury and pulmonary fibrosis, decreased RVH and total respiratory resistance, and enhanced RAC, pulmonary vascular density and pulmonary compliance, as well as inhibited EMT in airway epithelial cells in LPS and hyperoxia treated mice.

**Conclusion:**

Postnatal rhIGF-1/BP3 treatment relieved the effects of LPS or hyperoxia on lung injury and prevented RVH, providing a promising strategy for the treatment of BPD.

**Supplementary Information:**

The online version contains supplementary material available at 10.1186/s12890-023-02498-1.

## Introduction

Bronchopulmonary dysplasia (BPD) is a serious and long-term complication in premature infants that is caused by abnormal lung development. BPD occurs in more than 40% of preterm infants [1, 2]. In the United States, about 15,000 new cases are diagnosed every year [3]. BPD is closely associated with major complications, including prolonged mechanical ventilation, respiratory support and oxygen treatment, disordered angiogenesis, and a high incidence of advanced respiratory diseases [4]. If BPD is not well treated, many complications, such as mental retardation and pulmonary hypertension (PH), occur. More than 20% of infants with moderate to severe BPD develop PH. The incidence and mortality rates of BPD patients with pH have significantly increased [5]. Although significant advancements have been made in the fields of neonatal research and nursing [6, 7], the incidence and mortality rates associated with respiratory diseases in preterm infants remain high owing to the development of BPD. Therefore, effective preventive therapies are urgently needed.

Many prenatal factors, including chorioamnionitis (CA) and pulmonary edema (PE), are closely associated with an increased risk of BPD, especially those related to intrauterine growth [8, 9]. Insulin-like growth factor-1 (IGF-1), which has strong angiogenic activity, is implicated in the etiology of BPD [10]. Zhang et al. identified IGF-1 as a potential target for BPD treatment [11]. In addition, Han et al. investigated the relationship between serum IGF-1 levels and lung function [12]. However, whether treatment with IGF-1 improves respiratory diseases and prevents BPD remains unclear.

Based on the vital functions of IGF-1 in lung progression and reports stating that low IGF levels after birth are associated with the further development of BPD, our study aimed to (i) illustrate whether recombinant human IGF-1 (rhIGF-1)/binding peptide 3 (BP3) could prevent the development of PH in experimental BPD and (ii) uncover the latent mechanism of rhIGF-1/BP3 in vascular growth, lung structure, and lung function to identify promising therapies for BPD treatment.

### Materials and methods

### Animals

Pregnant Wistar rats (6–8 weeks, 200–250 g) were provided by Hubei Laboratory Animal Research Center (Wuhan, China) and housed under standard conditions (22–25 °C, 50–60% humidity) with free food and water. The rat pups were also housed under standard conditions (22–25 °C, 50–60% humidity) with free food and water. This study was conducted in accordance with the ARRIVE guidelines. All experimental protocols were in accordance with the National Institutes of Health Guide for the Care and Use of Laboratory Animals. The study protocols were approved by the Ethics Committee of the First Affiliated Hospital of Bengbu Medical College.

### Establishment of two models of BPD

BPD model of chorioamnionitis (CA): On day 20 of gestation, pregnant rats were randomly assigned to receive saline (control; 50 µl/sac) or LPS (10 µg/sac) stimulation [13]. Two days after the intra-amniotic injections, the neonatal rat pups were induced with rhIGF-1/IGFBP3 (0.2 mg/Kg/d, IP injections) or saline for 2 weeks. The rat pups were then sacrificed at 14 days of age for lung tissue collection for subsequent experiments.

Hyperoxia-induced BPD group (HOX group): Newborn rats were subjected to high oxygen tension (85% oxygen) or normoxic conditions (control) from days 1 through 14 of postnatal life [14]. Animals received rhIGF-1/BP3 (0.2 mg/Kg/d, IP injections) for 14 days. The pups were then killed at 14 days of age for lung tissue collection for subsequent experiments.

### Lung function detection

According to previous study [15], the lung function of 14-day old pups was determined using the flexiVent system (SCIREQ).

### Lung wet/dry weight (W/D) ratio measurement

The lung W/D ratio was evaluated to analyze the severity of the PE. Briefly, fresh lung samples were collected from 14 day old pups, blotted with filter paper, and weighed. After drying for 48 h at 65 ℃, lung samples were weighed again.

### Hematoxylin and eosin (H&E) staining

After the lung tissues were fixed with 4% paraformaldehyde for 24 h and dehydrated, slides with 5 μm paraffin sections were taken from the mid-plane of the left lower lobes of the fixed lungs. The sections were dewaxed, hydrated, incubated with hematoxylin for 5 min, and stained with eosin for 2 min, and the morphology of the lung tissue was observed using a microscope (×200 magnification).

### Masson staining

The 5 μm lung paraffin sections were dewaxed, hydrated, and stained using the Masson Staining Kit (G1006, Servicebio) according to the manufacturer’s protocol. The collagen fibers were stained blue, and the muscle fibers and red blood cells were stained red, indicating the content of collagen fibers and the degree of fibrosis in the tissue (×200 magnification).

### Evaluation of vascular density

Upper and lower lobe lung Sect. (5-µm-thick) were dissected, deparaffinized, rehydrated, and stained with von Willebrand factor (vWF; 1:100; Santa Cruz Biotechnology) and DAPI (D8417-1MG; Sigma). Subsequently, the number of vWF-positive vessels in each high-energy field (HPF) was observed using a blind method (×200 magnification).

### Immunofluorescence

Upper and lower lobe lung Sect. (5-µm-thick) were obtained and incubated with N-cad (1:200, 22018-1-AP, PTG), SP-C (1:100, DF6647, affinity), E-cad (1:200, 20874-1-AP, PTG), FSP1 (1:200, 20886-1-AP, PTG), and vim (1:100, 10366-1-AP, PTG) antibodies overnight at 4 °C. The next day, the sections were incubated with the secondary antibody for 2 h. Finally, fluorescence signals were visualized using a fluorescence microscope (×400 magnification).

### Right ventricular hypertrophy (RVH) evaluation

The right ventricle (RV) and left ventricle plus the ventricular septum (LV + S) were dissected and weighed. The RV/LV + S and RV/body weight ratios were then measured to analyze RVH.

### Evaluation of lung alveolarization

Slides with 5-µm paraffin sections were stained with H&E. Radial alveolar count (RAC) was used to evaluate alveolarization as previously described [16, 17].

### qRT-PCR analysis

Total RNA from lung tissues exposed to rhIGF-1/BP3 was harvested (EP013, ELK Biotechnology) and reverse-transcribed (Eq. 002, ELK Biotechnology). The relative mRNA levels of IGF-1 were determined using QuFast SYBR Green PCR Master Mix (Eq. 001, ELK Biotechnology) on StepOne™ Real-Time PCR (Life technologies). Target gene levels were analyzed using 2^−ΔΔCt^ method.

### Western blot analysis

Proteins were collected from the lung samples for western blot analysis of IGF-1 and eNOS using RIPA buffer (ASPEN, AS1004). Proteins were resolved using sodium dodecyl sulfate-polyacrylamide gel electrophoresis (SDS-PAGE) and transferred onto polyvinylidene difluoride (PVDF) membranes (Millipore, IPVH00010). The membranes were blocked with 5% nonfat milk and incubated with primary and secondary antibodies. Finally, the results were analyzed using ECL detection system reagents (ASPEN, AS1059) according to the manufacturer’s protocol.

### Statistical analysis

Statistical analyses were performed using the GraphPad Prism 8.0 software. Results are expressed as the mean ± SD from three independent measurements and analyzed using one-way analysis of variance (ANOVA) or Student’s t-test. Statistical significance was set at *P < 0.05 and **P < 0.01.

## Results

### Effects of rhIGF-1/BP3 on distal lung structure of experimental chorionic amniotic rats induced by LPS or high oxygen

**Fig. 1:**
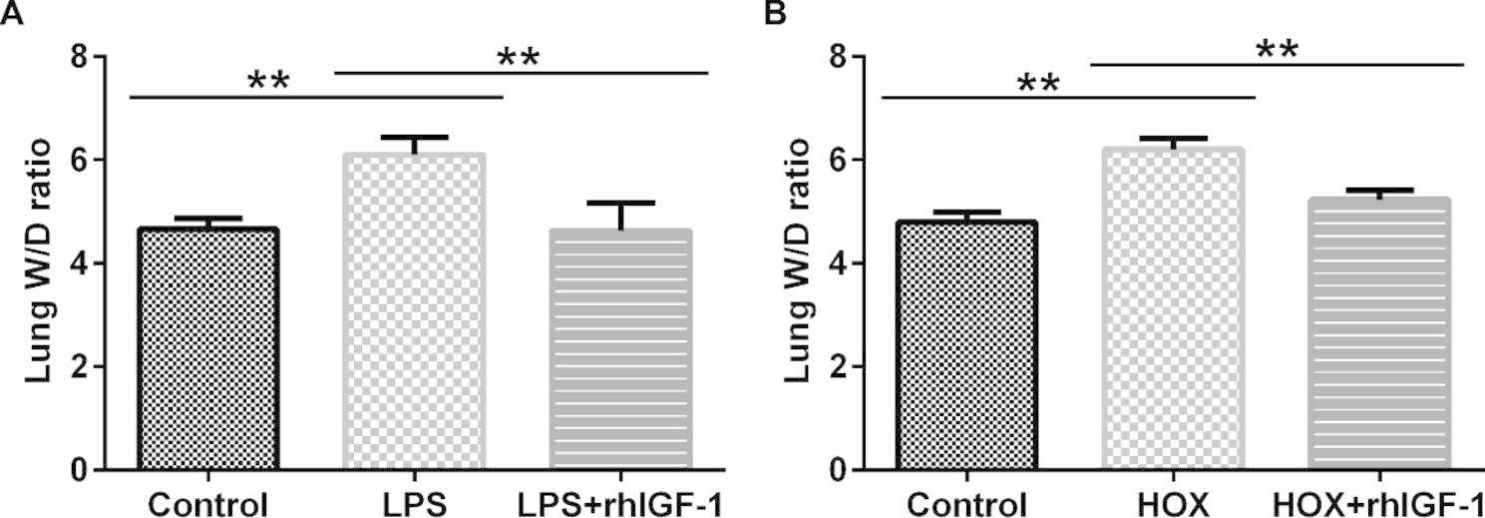
rhIGF-1/BP3 restored the dry/wet ratio to normal levels (A) The wet/dry weight ratio measured in the LPS + IGF, LPS, and Control groups. (B) The wet/dry ratio in the HOX + IGF, HOX and Control groups. n = 3; **P < 0.01

**Fig. 2:**
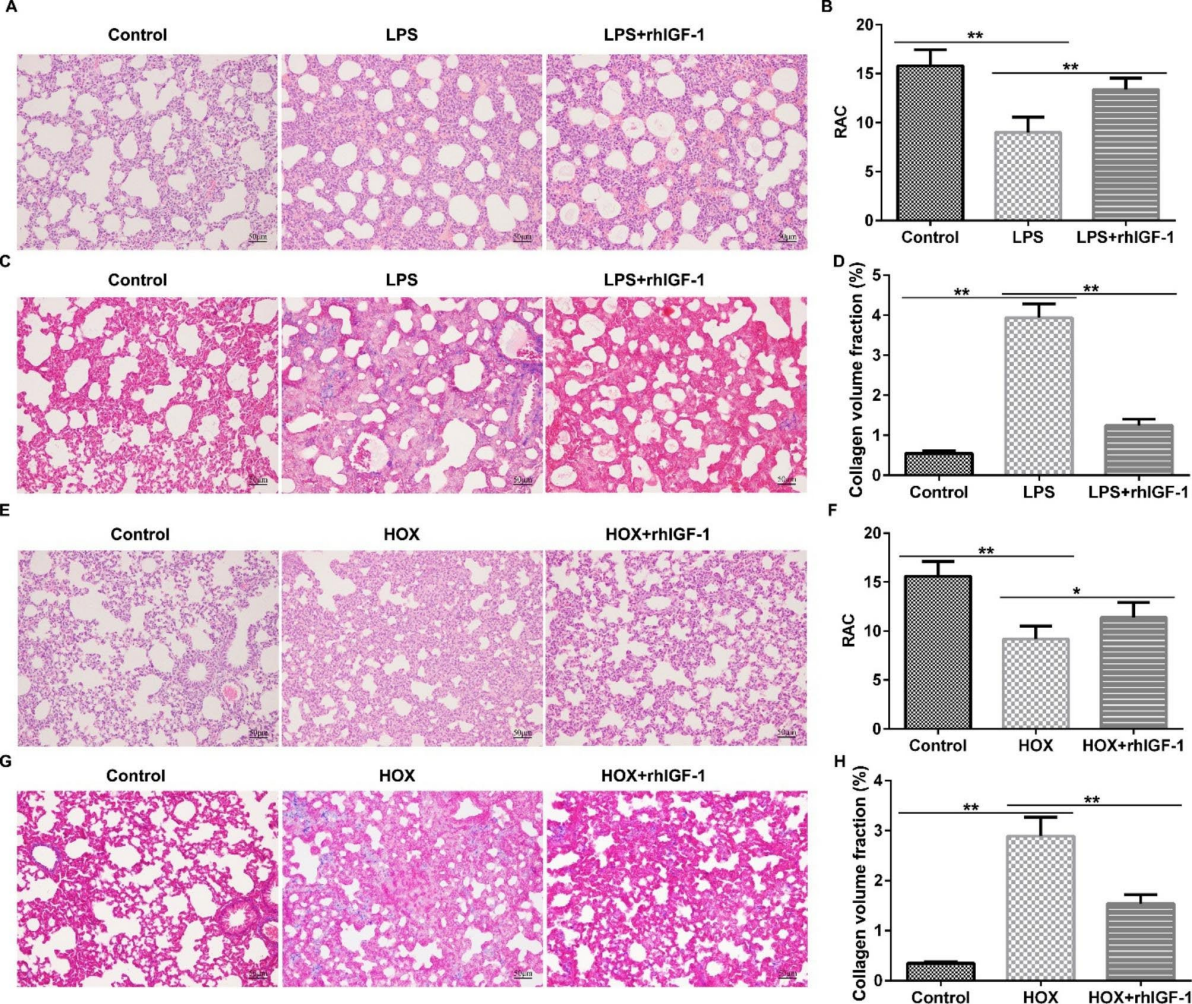
rhIGF-1/BP3 reversed the effects of LPS/high oxygen on distal lung structure of experimental chorionic amniotic rats (A and E) Representative pictures of HE staining in lung histology (n = 5). (B and F) Quantification of RAC numbers in different groups. (C and G) Representative pictures of Masson staining in pulmonary fibrosis (n = 3). (D and H) Quantitative images of collagen volume percentage. Magnification: ×200. * P < 0.05, **P < 0.01

To evaluate the influence of rhIGF-1/BP3 in postnatal BPD models, we investigated the role of rhIGF-1/BP3 in the distal lung structure of experimental chorionic amniotic rats after LPS or high-oxygen treatment. The lung W/D ratio in the LPS and high-oxygen treatment groups increased compared to that in the control group (Fig. 1), whereas the ratio decreased after rhIGF-1/BP3 treatment. We also observed that LPS stimulation caused a change in lung histology (Fig. 2A and B) and pulmonary fibrosis (Fig. 2C and D) in infant lungs, whereas we found the opposite results in rhIGF-1 treated groups. Moreover, similar findings were obtained in the high-oxygen-treated groups, as confirmed by the inhibition of RAC (Fig. 2E F) and promotion of collagen volume (Fig. 2G H), suggesting that rhIGF-1/BP3 treatment improves distal lung structure.

### Effects of rhIGF-1/BP3 on pulmonary vascular density of infant rats induced by LPS or high oxygen

**Fig. 3:**
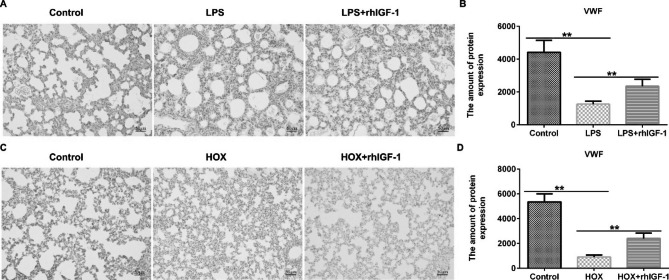
rhIGF-1/BP3 reversed the effects of LPS/high oxygen on lung vascular density in experimental chorioamnionitis. (A and C) Lung histology evaluated via immunostaining of endothelial cells with vWF. (B and D) vWF protein expression in different groups. Magnification: ×200. n = 3; * P < 0.05, **P < 0.01 vs

We also determined whether rhIGF-1/BP3 therapy could improve vessel density in infant rats. As shown in Fig. 3, vessel numbers after staining with vWF revealed that LPS treatment or high oxygen-induced rats had reduced vascular density compared to the control; however, rhIGF-1/BP3 stimulation caused the vascular density to reach normal levels. Our data revealed that rhIGF-1/BP3 increased pulmonary vascular density in infant rats, which was reduced by LPS or high oxygen levels.

### Effects of rhIGF-1/BP3 on right ventricular hypertrophy and pulmonary functions

**Fig. 4:**
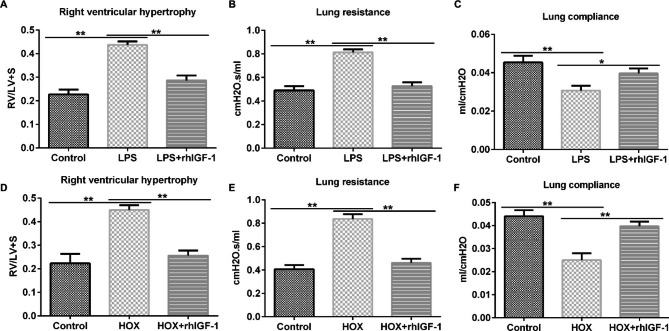
rhIGF-1/BP3 reversed the effects of LPS or high oxygen on RVH and lung function in infant rats rhIGF-1/BP3 stimulation improved RVH (A and D), lung resistance (B and E), and lung compliance (C and F) after LPS injection or high oxygen induction in experimental chorioamnionitis. n = 3; * P < 0.05, **P < 0.01

In addition to investigating the distal lung structure and vascular growth, we also observed that LPS stimulation enhanced RVH (Fig. 4A) and lung resistance (Fig. 4B) and suppressed lung compliance (Fig. 4C), which were reversed after rhIGF-1/BP3 treatment. Additional analysis of these indices revealed similar trends in RVH severity (Fig. 4D), lung resistance (Fig. 4E), and lung compliance (Fig. 4F) in the HOX groups. These findings demonstrate the repair function of rhIGF-1/BP3 in right ventricular hypertrophy and pulmonary function in infant rats.

### Effects of rhIGF-1/BP3 on LPS-induced EMT of airway epithelial cells in infant rats

**Fig. 5:**
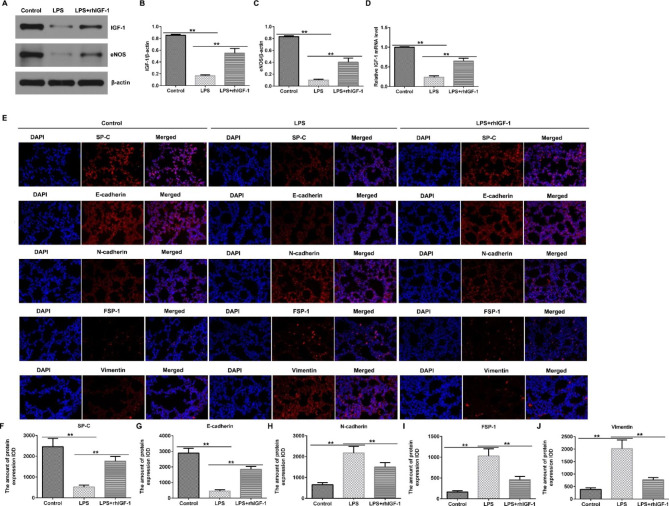
rhIGF-1/BP3 reversed the effects of LPS on airway epithelial cells EMT in infant rat in experimental chorioamnionitis (A) Western blot analysis of IGF-1 and eNOS expression. (B-C) Relative expression of IGF-1 and eNOS. (D) mRNA levels of IGF-1 evaluated using qRT-PCR. (E) Representative images of alveolar epithelial and mesenchymal cell marker immunofluorescence. (F-J) Relative expression of SP-C, E-cadherin, N-cadherin, FSP1, and Vimentin. Magnification: ×400. n = 3; * P < 0.05, **P < 0.01

To further explain the mechanisms associated with the effects of rhIGF-1/BP3 on lung structure in these models, we evaluated the role of rhIGF-1/BP3 in the prevention of changes in pH and EMT in airway epithelial cells. We observed reduced IGF-1 mRNA level and protein expression and reduced eNOS protein levels after LPS exposure (Fig. 5A and D). Immunofluorescence was used to detect alveolar epithelial and mesenchymal cell markers including SP-C, E-cadherin, N-cadherin, FSP1 and Vimentin. LPS induction reduced alveolar epithelial cells and promoted the EMT of airway epithelial cells, whereas we found the opposite results in the rhIGF-1/BP3 treated groups (Fig. 5E J).

### Effects of rhIGF-1/BP3 on hyperoxia-induced EMT of airway epithelial cells in infant rats

**Fig. 6:**
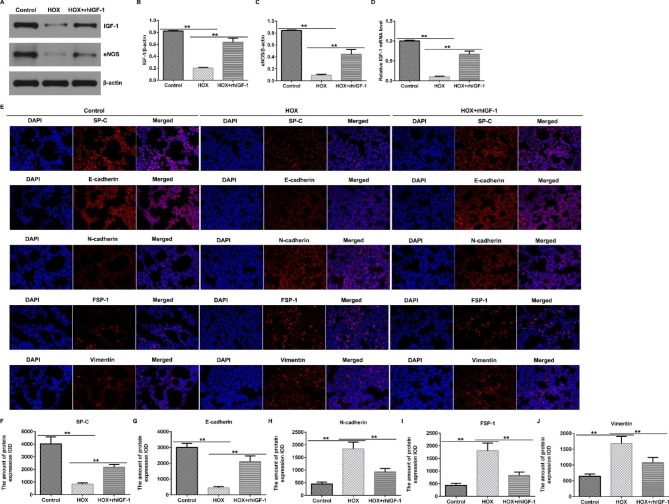
rhIGF-1/BP3 reversed the effects of high oxygen on airway epithelial cells EMT in infant rat in experimental chorioamnionitis (A) Detection of IGF-1 and eNOS expression using western blotting. (B-C) Relative expression of IGF-1 and eNOS. (D) qRT-PCR analysis of IGF-1 mRNA levels. (E) Alveolar epithelial and mesenchymal cell markers identified using immunofluorescence. (F-J) Relative expression of SP-C, E-cadherin, N-cadherin, FSP1, and Vimentin. Magnification: ×400. n = 3; * P < 0.05, **P < 0.01

We also determined whether rhIGF-1/BP3 treatment plays a vital role in the prevention of changes in pH and EMT in airway epithelial cells in BPD models stimulated with hyperoxia. Western blotting and qRT-PCR demonstrated that hyperoxia induction suppressed IGF-1 mRNA level and protein expression, and decreased eNOS protein levels (Fig. 6A and D). Immunofluorescence findings were similar to those of LPS treatment, as evidenced by reduced SP-C and E-cadherin levels and increased N-cadherin, FSP1 and Vimentin expression (Fig. 6E J); however, rhIGF-1/BP3 treatment reversed the effects of hyperoxia on EMT in airway epithelial cells in infant rats. Our data suggest that rhIGF-1/BP3 improves BPD progression by preventing PH and inhibiting EMT in airway epithelial cells.

## Discussion

BPD, a chronic lung disease caused by abnormal lung development, is common in premature infants and is characterized by the destruction of pulmonary angiogenesis [18, 19]. Approximately 30% of infants will develop PH, which is one of the major causes of the high incidence rate and mortality in BPD. The pathophysiology of PH in patients with BPD includes pulmonary vascular reduction, endothelial cell dysfunction, increased vascular tension, and altered vascular reactivity [20]. Therapeutic therapies for endothelial cell function and survival may help develop new methods for the prevention of BPD. Papagianis et al. have confirmed the potential anti-inflammatory therapies for BPD [6], and Tin et al. have reported potential drug therapies for BPD [21]. However, the specific mechanism by which placental dysfunction affects lung disease progression and leads to BPD remains unclear.

In vitro investigations have revealed that developing endothelial cells are vital for the regulation of epithelial growth by producing key factors, including hepatocyte growth factor and IGF-1 [22]. IGF-1 plays an important role in fetal growth and organogenesis by regulating cell growth, maturation, and differentiation. 21. Schmidt et al. confirmed that IGF-1 treatment improves outcomes in rats with cancer cachexia [23]. Seedorf et al. reported that rhIGF-1/BP3 injections significantly reduced BPD and increased IGF-1 levels in preterm infants [13]. Based on these findings, we hypothesized that the early restoration of IGF-1 levels may play a protective role against severe retinopathy in premature infants. Therefore, this study aimed to evaluate whether postnatal treatment with rhIGF-1/BP3 could prevent PH in BPD.

Several factors are involved in the pathogenesis of BPD, including hyperoxia and mechanical ventilation [24, 25]. In our study, we generated two models of BPD to investigate the underlying mechanism of rhIGF-1/BP3 in the progression of BPD: one model was associated with CA induced by intra-amniotic fluid and exposure to LPS, and the other was exposed to postnatal hyperoxia. The newborn rats were then injected with rhIGF-1/BP3 (0.2 mg/Kg/d) or saline. Previous studies have suggested that exposure to hyperoxia may damage the alveolarization and pulmonary vascularization in preterm infants [26]. First, we evaluated the degree of lung histopathology and pulmonary fibrosis using H&E and Masson staining of the lower lobe of the left lung. Our findings are in accordance with other reports suggesting that rhIGF-1/BP3 improves LPS-induced changes in lung histology, pulmonary fibrosis, and increased collagen volume in infant lungs. RAC was used to assess the effect of rhIGF-1/BP3 on lung tissue injury in rats with BPD. We found that RAC was inhibited, and similar findings were obtained in the high oxygen-treated groups, suggesting that rhIGF-1/BP3 treatment improves distal lung structure in BPD rats.

Adverse prenatal elements such as chorioamnionitis, PE, or postpartum damage may result in pulmonary vascular disease, which not only causes PH but also affects distal lung growth [27]. Therefore, we determined the effects of rhIGF-1/BP3 on the vessel density in infant rats. Our findings revealed that LPS treatment or high-oxygen stimulation decreased vessel density; however, rhIGF-1/BP3 stimulation restored vascular density to normal levels. It should be mentioned that we measured the vascular density through vWF staining, and as it is the localization of extracellular matrix and secreted proteins, there will definitely be non-specific staining. During the analysis process, we can only try to narrow the selection range and avoid including too many non-specific staining in the statistical range. This was a limitation of the current study. We also assessed the effects of rhIGF-1/BP3 on right ventricular hypertrophy and pulmonary function by determining RVH, lung resistance, and lung compliance. Our findings show that rhIGF-1/BP3 therapy preserves the lung structure and function and prevents RVH in rat pups. Therefore, in the two models of BPD, rhIGF-1/BP3 treatment played a significant role in normalizing lung function and preventing RVH in infant rats, which further supports previous research that has identified IGF-1 as a critical regulator of lung development.

To further explain the mechanisms related to rhIGF-1/BP3 in the lung structure of these models, we detected the expression of IGF-1 or eNOS in the lungs using western blotting. We observed reduced IGF-1 mRNA level and protein expression and reduced eNOS protein levels after LPS or hyperoxia exposure. Moreover, we assessed the alveolar epithelial and mesenchymal cell markers in the lungs of BPD rats. Immunofluorescence revealed that LPS induction or hyperoxia exposure reduced the number of alveolar epithelial cells and promoted the EMT of airway epithelial cells, whereas we found the opposite results in the rhIGF-1/BP3 treated groups. However, this study did not elucidate the specific molecular regulatory mechanism of rhIGF-1/BP3 in regulating EMT, which is another limitation of this study.

In conclusion, our results revealed that rhIGF-1/BP3 treatment accelerated lung angiogenesis, improved lung structure and function, and prevented the development of RVH in two experimental BPD models. Our study suggests a promising therapy for BPD in preterm infants. Following these achievements in BPD research, additional antenatal models that result in impaired lung development need to be further investigated.

## Electronic supplementary material

Below is the link to the electronic supplementary material.

Supplementary Material 1

## Data Availability

The datasets used and/or analyzed during the present study are available from the corresponding author upon reasonable request.

## Abbreviations

ANOVA: Analysis of variance
BP3: Binding peptide 3
BPD: Bronchopulmonary dysplasia
CA: Chorioamnionitis (CA)
H&E: Hematoxylin and eosin
HPF: High-energy field
HOX group: Hyperoxia-induced BPD group
IGF-1: Insulin-like growth factor 1
LV + S: Left ventricle plus the ventricular septum
PVDF: Polyvinylidene difluoride
PE: Pulmonary edema
PH: Pulmonary hypertension
RAC: Radial alveolar count
rhIGF-1: Recombinant human IGF-1
RV: Right ventricle
RVH: Right ventricular hypertrophy
SDS-PAGE: Sodium dodecyl sulfate-polyacrylamide gel electrophoresis
vWF: Von Willebrand factor
W/D: Wet/dry weight
