# Supplementary figures and images for: The role of rhIGF-1/BP3 in the prevention of pulmonary hypertension in bronchopulmonary dysplasia and its underlying mechanism
Source: BMC Pulm Med. 2023 Jun 15;23:209. doi: 10.1186/s12890-023-02498-1 (PMC10273711; doi:10.1186/s12890-023-02498-1)

**Figure 4**

A1: Control; B1: LPS; C1: LPS+rhIGF-1


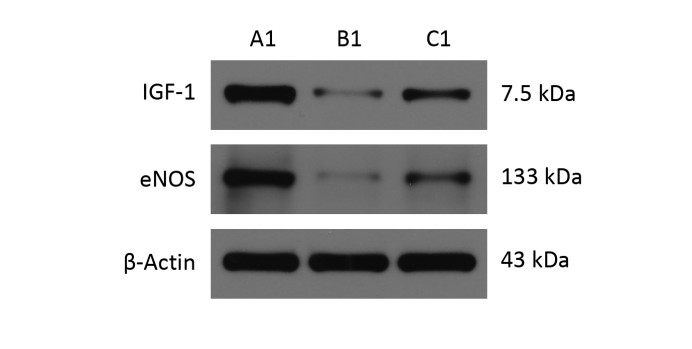


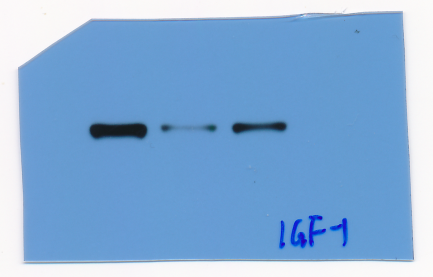


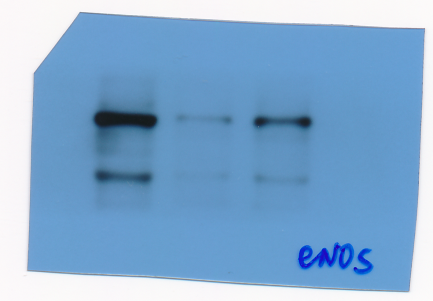


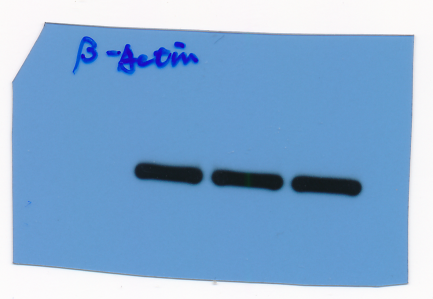


**Figure 5**

A12: Control; B2: HOX; C2: HOX+rhIGF-1


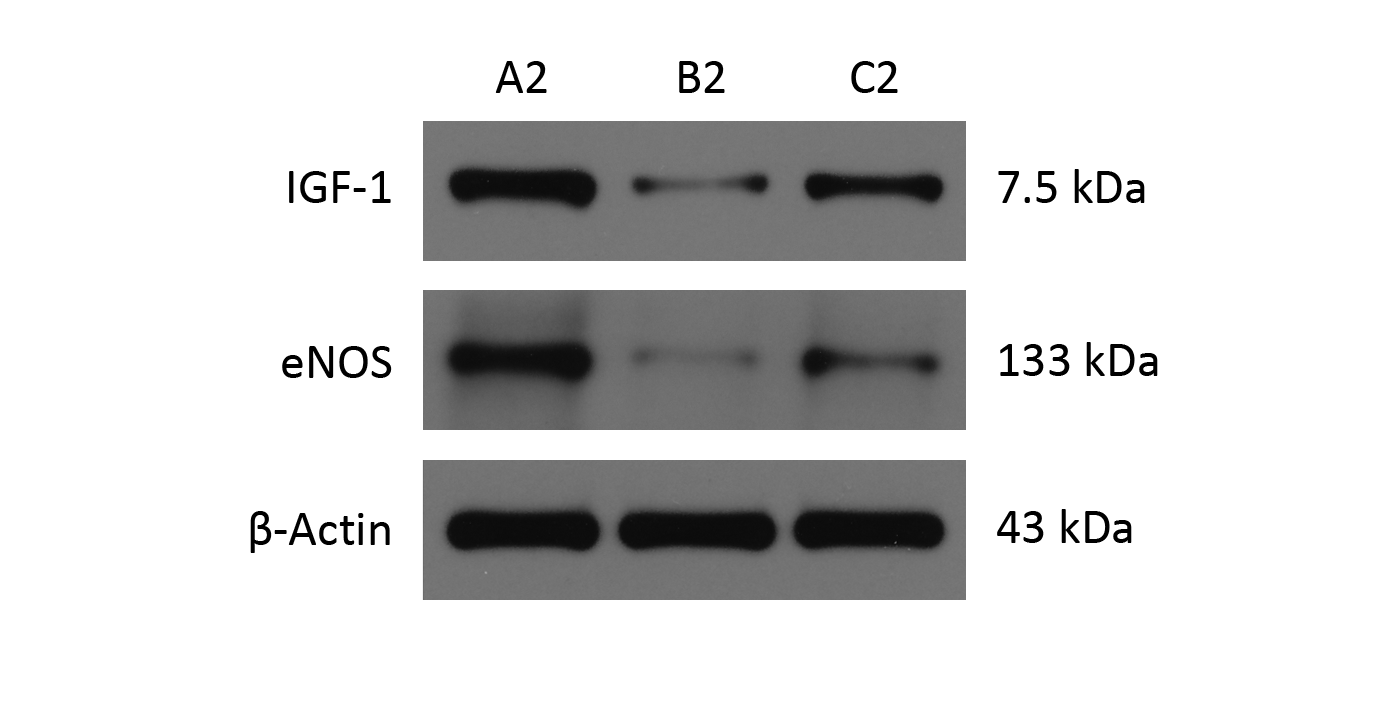


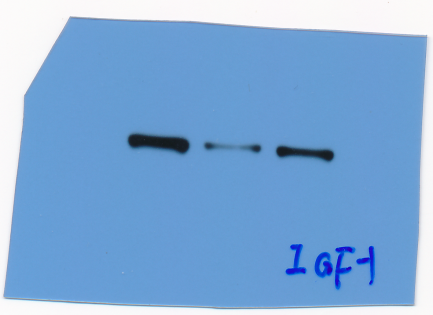


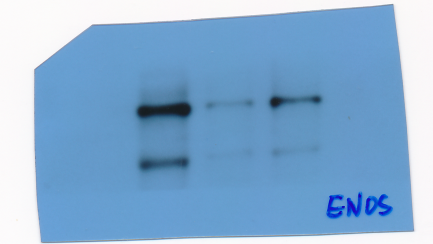


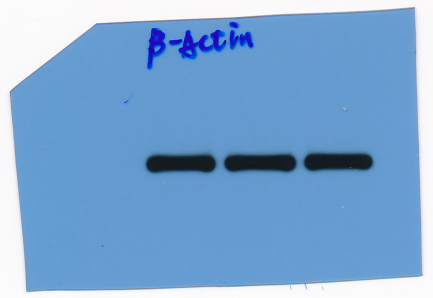

Supplement: Supplementary file 1 — Supplementary Material 1 [file 12890_2023_2498_MOESM1_ESM.docx]
